# The “Wear and Tear” of the Organism in Temporomandibular Disorders: A Pilot Study Investigating the Effects of Allostatic Load on Heart Rate Variability and Inhibitory Control

**DOI:** 10.3390/jpm14080787

**Published:** 2024-07-25

**Authors:** Giovanna Troisi, Paola Di Giacomo, Giuseppe Forte, Viviana Langher, Maria Casagrande, Carlo Di Paolo

**Affiliations:** 1 Department of Psychology, University of Rome “Sapienza”, 00185 Rome, Italy; 2 Department of Experimental Psychology, Mind, Brain, and Behavior Research Center (CIMCYC), University of Granada, 18071 Granada, Spain; 3 Department of Oral and Maxillo-Facial Sciences, Policlinico Umberto I, 00161 Rome, Italy; 4 Department of Dynamic, Clinical Psychology and Health Studies, University of Rome “Sapienza”, 00185 Rome, Italy

**Keywords:** chronic pain, temporomandibular disorders, allostatic load, heart rate variability, inhibitory control

## Abstract

Temporomandibular disorders (TMDs) are the most common cause of non-dental chronic pain in the orofacial region and can chronically increase the activity of the allostatic systems. The allostatic overload related to these conditions causes an autonomic dysregulation, reflected by a reduction in heart rate variability (HRV). Nevertheless, chronic pain in these patients could cause more severe health consequences, such as those related to cognitive functioning. Deficits in executive control have been associated with allostatic overload and could negatively affect pain management strategies. This study aimed to investigate the effects of chronic pain on HRV and both motor and cognitive inhibition (assessed with the Go/No-Go and Stroop tasks, respectively) in a sample of 14 patients with TMD and 15 healthy controls. Consistent with our hypothesis and the previous literature, the group with TMD had a lower resting HRV, but no differences were found between the groups in inhibition. Furthermore, the results showed that the effects of HRV on cognitive inhibition can be mediated by pain intensity. Finally, a correlation between age and HRV emerged in patients with TMD but not in healthy controls.

## 1. Introduction

Temporomandibular disorders (TMDs)—a group of musculoskeletal dysfunctions [1]—are the most common cause of non-odontogenic chronic pain in the orofacial region [2]. Research suggests that within a multifactorial etiology, biopsychosocial factors contribute to the onset, severity, and duration of the symptoms [3].

TMD-related pain is closely associated with structural and functional alterations in the neural mechanisms involved in pain modulation [4]. Acute pain elicits the concerted activation of multiple systems, i.e., the hypothalamic–pituitary–adrenal axis, the metabolic systems, the autonomic nervous system (ANS), and the immune system. This active process of maintaining or restoring homeostasis has been defined as “allostasis” [5].

Due to its persistent and prolonged nature, chronic pain can lead to the overactivity of allostatic systems, resulting in “allostatic overload” [6,7] which can expose the organism to stress-related diseases [8,9].

With this in mind, research has attempted to identify useful and reliable markers of the allostatic load in order to prevent potential adverse outcomes. For this purpose, high heart rate variability (HRV), a measure of autonomic activity [10,11,12], has been widely considered an index of self-regulation abilities and has been increasingly linked to cognitive functions [13,14]. Specifically, a higher HRV is associated with better executive functions, attention, and working memory [13,15,16]. This relationship is primarily attributed to the influence of the vagus nerve, which influences both cardiac function and prefrontal cortex activity. Consequently, HRV has been proposed as a potential biomarker of cognitive health [17,18].

Pain elicits an increase in sympathetic activity, which allows the organism to prepare an adaptive response [19,20,21]; nevertheless, chronic pain can persistently increase sympathetic activity, resulting in an autonomic imbalance and a reduced HRV. According to allostatic load theory and experimental evidence, TMDs have been associated with a generalized impairment of the ANS [22,23], with reduced HRV in patients with temporomandibular disorders compared to healthy controls, even during relaxation [24].

This “wear and tear” on the organism occurs primarily at the neurophysiological level, but it can degenerate into more severe health outcomes, such as a decrease in global cognition and executive functions [25].

This evidence can be relevant for its clinical implications; in fact, inhibition—a core component of executive functions that allow for silencing irrelevant stimuli during goal-directed behaviors [26,27,28]—has been related to pain perception in healthy individuals [29,30,31,32,33].

Moreover, studies have suggested altered inhibitory control in patients with chronic pain [34], such as TMDs [35,36], but findings are contrasting; patients with TMDs have shown slower responses [36] and different patterns of brain responses during the Stroop task compared to controls, underlining the improper activity of emotional networks. Nevertheless, Hollins et al. [37] found stronger cognitive inhibition in patients with a history of chronic pain compared to controls, probably due to the development of inhibition strategies for pain management.

According to the aforementioned evidence and the allostatic load framework, this study aimed to assess differences in HRV and inhibitory control between healthy individuals and patients with TMD. We hypothesized that patients with TMD would show reduced HRV at rest compared to controls. Furthermore, despite the inconsistent results regarding inhibition abilities in chronic conditions, we hypothesized that patients with TMD—who are thought to be more familiar with inhibiting irrelevant stimuli during their activities—would show stronger cognitive and motor inhibition compared to controls.

In light of the potential relationship between pain intensity and HRV, which may be attributed to allostatic imbalance, we hypothesized that an independent and synergistic interaction could predict cognitive performance.

## 2. Materials and Methods

### 2.1. Participants

This pilot study included 15 patients diagnosed with TMD and 15 healthy subjects matched for sex and age; nevertheless, HRV data from one participant were unavailable due to a problem during the HRV recording. Thus, the final sample included 14 patients (11 women, 3 men) and 15 healthy subjects (12 women, 3 men). The sample size (*n* = 30) was calculated using a pre-established level of confidence (0.80) and a given probability (0.05), adopting a specific formula designed for pilot studies [38]. The descriptive statistics of each group are synthesized in Table 1.

All participants took part in this study voluntarily. Healthy controls were recruited through flyers published on the Health Psychology Laboratory website. The flyers provided information regarding the inclusion criteria (e.g., being aged above 18 years and being free from chronic clinical/psychiatric conditions).

Patients were recruited at the Department of Oral and Maxillofacial Sciences of the Policlinico Umberto I, Sapienza University of Rome. The diagnosis of TMDs was assessed by an expert and calibrated staff according to the Diagnostic Criteria for TMD (DC/TMD). Patients treated at the polyclinic who met the inclusion criteria were approached by the clinicians and asked if they would voluntarily participate in this study. If they agreed, they were contacted in order to schedule an appointment for the experimental procedure. Inclusion criteria for patients were the following: (1) diagnosis of TMD; (2) not following a pharmacological treatment for pain management; (3) being over 18 years of age; (4) experiencing chronic pain intensity above 40 on the Verbal Numeric Scale (VNS) for a period of at least 6 months.

Participants (both patients and healthy controls) were excluded if the clinical history was positive for head trauma, psychiatric disorders, and/or other clinical conditions (e.g., cardiovascular and respiratory diseases, hormonal disorders) that could influence HRV measurement and cognitive performance. All participants signed a written informed consent, according to the Declaration of Helsinki. The study protocol was approved by the Local Ethical Committee of the University of Rome “Sapienza” (Prot. no. 0001168, Department of Dynamic and Clinical Psychology and Health).

### 2.2. Measures

To collect sociodemographic and anamnestic data, a face-to-face semi-structured interview was adopted. The anamnestic interview also assessed inclusion and exclusion criteria.

#### 2.2.1. Pain Intensity

In order to evaluate the intensity of perceived chronic pain recently, participants rated the intensity of pain experienced in the last two weeks prior to the evaluation on a Numerical Rating Scale (NRS), ranging from 0 (i.e., no pain) to 10 (i.e., unbearable pain).

#### 2.2.2. Heart Rate Variability

HRV was recorded using a Firstbeat Bodyguard-2 (Firstbeat Analytics, Jyvaskyla, Finland). The signal was processed using Kubios HRV Analysis 3.4.3 software [39]. In particular, we evaluated the HRV by considering both frequency-domain and time-domain indices of vagal activity [40]. The frequency-domain analysis provided the mean spectral power measures of the high-frequency band (HF-HRV, 0.15–0.4 Hz); the time-domain analysis considered the root mean square of successive standard deviation (RMSSD). Furthermore, the standard deviation of the mean RR interval (SDNN) was included in the analysis as reflective of the variability in the recording period. RMSSD is considered a robust marker of parasympathetic nervous system activity. Similar to RMSSD, HF power reflects parasympathetic nervous system activity. Higher RMSSD and HF values generally indicate greater parasympathetic activity and better autonomic function. Higher SDNN suggests a balance between the sympathetic and parasympathetic nervous systems. We evaluated HRV during a baseline condition (resting). HRV indices were measured in ms^2^ and then normalized using natural logarithms in order to reduce the variability in the data [41].

#### 2.2.3. Cognitive Measures

##### Stroop Task

A computerized version of the Stroop task [42] provided the administration of colored words (font: Courier New; font size: 60; colors: yellow, red, blue, green) semantically related to the colors YELLOW, RED, BLUE, GREEN. Each word could be presented in a (i) congruent condition, with the ink color related to its semantic meaning (e.g., BLUE written in blue ink), or in an (ii) incongruent condition, where the ink color was related to the semantic meaning of another color (e.g., BLUE written in red ink). Participants were required to press the key corresponding to the ink color (key “A” = red; key “S” = green; key “K” = blue; key “L” = yellow) as quickly and accurately as possible. At the beginning of the task, a practice block of 15 trials was presented with feedback on correct performance. Afterward, a block of 120 randomly presented trials (60 congruent and 60 incongruent) was presented. An initial fixation cross (duration: 400 ms) was presented before each trial. The target stimulus duration was 3000 ms or until the participant’s response. Reaction times and accuracy on the task were collected. Trials with reaction times greater than 200 ms and correct trials were considered to calculate each participant’s mean reaction times (RTs) for the congruent and incongruent conditions, and the Stroop effect (mean RT incongruent trials—mean RT congruent trials) was computed.

##### Go/No-Go Task

Two geometric shapes (960 × 720 pixels) appeared individually in the center of the screen with a black background. The Go (target) stimulus was a green circle, and the No-Go (non-target) stimulus was a green triangle. An initial screen with a fixation cross (duration: 500 ms) was followed by the randomized presentation of the stimuli. Each stimulus remained in the center of the screen for 750 ms or until the participant’s response. Participants were required to press the left mouse key as quickly as possible when the green circle appeared in the center of the screen; when the green triangle appeared, the participant had to wait for the stimulus to disappear. A total of 100 trials, divided into two blocks of 50 trials each, were administered. A practice block of 12 trials, with feedback on correctness, was presented at the beginning of the experiment. Incorrect responses to No-Go stimuli were summed to define the number of false alarms, which was considered to measure the inhibition motor component.

### 2.3. Procedure

Each participant underwent a single experimental session. At the beginning of the session, the written informed consent was signed, and the anamnestic data were collected. All participants were asked if they had suffered from pain in the last two weeks in order to control for any possible pain-related conditions in the control group. Participants in the control group did not report any persistent pain experiences in the two weeks prior to the experimental session. The NRS for the evaluation of pain intensity was administered to patients with TMD. Participants were asked to rate the intensity of the pain experienced in the past two weeks. Then, the ECG recording device was placed with one sensor attached under the right collarbone and the other under the left rib cage. Resting HRV (i.e., participants were instructed to relax on a comfortable chair) was collected for 5 min; thereafter, the device was removed.

Then, participants completed the Stroop task (five minutes) and the Go/No-Go Task (five minutes) in a counterbalanced order. At this point, the experimental procedure was concluded. The whole procedure took about 20 min.

### 2.4. Data Analysis

Statistical analyses were computed using Jamovi 2.3.18 software (Sydney, Australia), with significance set at *p* < 0.05. According to the aim of this study, independent sample *t*-test analyses were conducted to highlight differences between groups (i.e., patients with TMD, healthy controls) in HRV indices and the performance on inhibition tasks.

According to the literature, age could influence HRV, with adults showing lower resting vagal tone beginning in the third decade of life [43]; nevertheless, some evidence suggests that HRV could be impaired only in adults affected by clinical, psychiatric, and chronic conditions [44,45,46]. To clarify the relationship between age and HRV levels, two separate Pearson’s correlations were studied in the two groups.

Furthermore, regression models considering HRV and pain intensity as predictors of cognitive functions, were carried out in order to assess the independent synergistic role of pain intensity and HRV on cognitive performance.

## 3. Results

### 3.1. Descriptive Statistics

The descriptive statistics are summarized in Table 1.

**Table 1:** Descriptive statistics and independent sample *t*-test analyses.

*n* = 29	TMD (*n* = 14)		Control (*n* = 15)		*t*	*p*
	*n*	M (SD)	*n*	M (SD)		
**Sex (F/M)**	11/3		12/3			
**Age**		42 (13.82)		41.53 (12.42)		
***HRV measures* ^1^**						
**SDNN**		3.39 (0.351)		3.59 (0.405)	−1.309	*0.102*
**RMSSD**		2.99 (0.412)		3.36 (0.500)	**−2.055**	0.026 *
**HF**		4.80 (0.944)		5.75 (0.929)	**−2.743**	0.005 **
***Cognitive performance* ^2^**						
**Stroop Effect**		75.498 (74.462)		72.261 (34.413)	0.152	*0.880*
**Go/No-Go—False Alarms**		2.64 (4.534)		3.54 (2.989)	−0.601	*0.553*
***Pain Intensity***						
**NRS**		2.44 (3.16)				

TMD: temporomandibular disorder; SDNN: standard deviation of all normal RR (NN) intervals; RMSSD: root mean square of successive differences between normal heartbeats; HF: high frequency; NRS: Numerical Rating Scale. HRV indices were transformed in natural logarithms. ^1^ The alternative hypothesis specified that TMD group < control group. ^2^ The alternative hypothesis assumed that TMD group ≠ control group. * *p* < 0.05; ** *p* < 0.01.

### 3.2. t-Test for Independent Samples: HRV Measurements

The *t*-test for independent samples analyzed the differences between the groups in the HRV indices (i.e., SDNN, RMSSD, HF), and the results confirmed significantly lower HRV measures in patients with TMD than in the control group; specifically, RMSSD (df = 23.0; *t* = −2.06; *p* = 0.026) and HF (df = 27.0; *t* = −2.74; *p* = 0.005) were significantly lower in patients with TMD than in the control group (see Table 1).

### 3.3. t-Test for Independent Samples: Inhibition Measurements

The *t*-test for independent samples analyzed the differences between the groups in their performance on both the Stroop (i.e., Stroop effect) and the Go/No-Go (i.e., false alarms) tasks; the results revealed no significant differences between groups in either cognitive or motor inhibition (Table 1).

### 3.4. Correlations between Age and HRV Indices

Pearson’s correlations were performed to analyze the relationship between age and HRV measures in both groups (Table 2). The results revealed significant negative correlations between age and HRV measures (SDNN: r = −0.71, *p* = 0.002; RMSSD: r = −0.57, *p* = 0.02; HF: r = −0.54, *p* = 0.03) in patients with TMD but not in the control group (SDNN: r = −0.36, *p* = 0.19; RMSSD: r = −0.30, *p* = 0.27; HF: r = −0.38, *p* = 0.16).

### 3.5. Regression Model

Two regression models (Table 3) were performed considering cognitive performance (i.e., Stroop effect and false alarms) as the dependent variables, with the natural logarithm of RMSSD and pain intensity (i.e., scores on the NRS) as predictors. The results showed a relationship between HRV, pain intensity, and the Stroop effect (R^2^: 0.82; Adj. R^2^: 0.78; *p* < 0.001). Pain intensity (*p* = 0.008) and HRV (*p* = 0.003) exhibited an independent predictive ability, whereas the synergistic interaction (*p* = 0.23) did not. No significant effects were found for false alarms.

## 4. Discussion

Chronic pain in patients with TMD can challenge their allostatic systems, leading to the phenomenon of “allostatic overload”. Given the potential negative consequences of the allostatic system overload, the aim of this study was to assess differences in autonomic activity and both motor and cognitive inhibition in patients with TMD and healthy controls. Regarding autonomic measures, we hypothesized that the patients would have a lower HRV than the healthy individuals; the results were consistent with previous findings [47] and confirmed our hypothesis. Furthermore, we found that lower levels of HRV were negatively associated with age in patients with TMD but not in healthy controls; in line with our hypothesis, this result could suggest that an imbalance in autonomic activity could be used to discriminate between healthy adults from those suffering from diseases that impair self-regulation abilities.

Previously, some researchers have questioned whether autonomic imbalance was only a consequence of the chronic condition or if it played a critical role in its onset and persistence. Several studies have highlighted that acute pain elicits a strong sympathetic response [10]; during acute pain stimulation, sympathetic activity increases, and parasympathetic activity decreases in order to recruit the physiological systems necessary for pain management.

Vagal tank theory [12] posits that autonomic responses can change in three distinct phases known as the three “Rs”: rest, reactivity, and recovery. Rest is a state characterized by the absence of a stressor, and autonomic balance is primarily controlled by parasympathetic activity. During the reactivity phase, the organism is required to respond to an internal or environmental stressor; in this case, the organism may exhibit either lower or higher vagal withdrawal depending on the demands of the task. Once the stressor has been faced, the organism initiates the recovery phase, during which vagal HRV indices gradually return to the baseline in a restoration process.

In chronic pain conditions, such as TMDs, the exposure to frequent and prolonged stress may result in elevated physiological arousal [48]. This impaired pattern gives rise to an imbalance in baseline HRV measurements, as evidenced by the findings of this study. The aforementioned mechanisms are consistent with the allostatic load framework, which highlights a decrease in vagal flexibility in patients with TMDs, even under resting periods.

Moreover, our results did not confirm the second hypothesis of this study regarding motor and cognitive inhibition abilities. In fact, there were no significant differences between patients and controls, suggesting that both motor and cognitive inhibition abilities are similar in the two groups and remain unimpaired in TMDs. Nevertheless, the regression model suggested that pain intensity and autonomic dysregulation independently predict cognitive performance. These findings could be explained in terms of allostatic load and are supported by previous evidence.

First, only the behavioral component of inhibition was considered in this study. A recent study by Glass et al. [49] investigated both motor and cognitive inhibition abilities in patients affected by fibromyalgia and did not find any behavioral differences in the performance between the two groups; nevertheless, a different pattern of neural activation during the inhibition tasks was observed in patients, with an over-involvement of premotor areas compared to healthy individuals. These results could suggest that when faced with chronic stress, the brain recruits a great number of cognitive resources to process and cope with an adverse stimulus; for this reason, the limited cognitive resources available are reorganized in order to respond efficiently to the demands of the task.

Furthermore, some researchers have postulated that the imbalance resulting in allostatic overload can be reflected by a series of outcomes that occur in a cascade effect [25]: the primary outcome of overactivation involves neurophysiological systems; the second outcome involves more complex mechanisms, such as the cardiovascular system; the third class of effects are the health outcomes that affect multiple domains, such as cognition [25,50].

From this perspective, it is reasonable to assume that the TMD-related symptoms of our sample were not severe enough to compromise different levels of functioning; for this reason, the allostatic load involved only the first class of outcomes but not superior cognitive functions. This study has some limitations: First, the sample size was small because it was a pilot study; an increase of the number of participants could influence the results, and could also allow researchers to divide the sample according to the specific type of TMD in order to provide more information regarding each specific condition involving the temporomandibular area. The second limitation attributable to the sample is in regards to the prevalence of women participants; nevertheless, this proportion reflects the prevalence of the TMDs that are generally more frequent among women than men. Moreover, the age range was very wide for a small sample like ours; this aspect could have influenced the results because both autonomic [51] and cognitive functions can change across the lifespan [28]. Further research could focus on specific age ranges in order to reduce the influence of age on the results. Finally, a limitation could be related to the rates of pain intensity; in fact, the patients involved in this study reported low levels of pain experienced in the two weeks before the experimental session. Further differences between participants experiencing different levels of pain intensity could be investigated in a larger sample.

## 5. Conclusions

HRV measures are reliable in assessing autonomic imbalance in TMDs. The allostatic load due to these chronic conditions was found to affect physiological systems but not the behavioral component of both motor and cognitive inhibition since no differences were found between the two groups. Further research is needed to investigate the inhibition abilities and physiological patterns related to inhibitory control in order to improve clinical outcomes in patients with TMDs.

Despite some limitations, this study provides important insights into the changes that occur in patients with TMDs. In fact, while the presence of autonomic alterations in these chronic conditions is well established, research on inhibitory abilities in chronic pain populations is still lacking. Future research should focus on inhibition abilities in populations with chronic pain in order to provide support to clinical interventions aimed at improving pain management strategies. The effectiveness of these interventions could ameliorate the well-being of populations suffering from chronic pain and help them to maintain their functional abilities in daily life.

## Figures and Tables

**Table 2:** Correlations between HRV indices and age.

	Group		SDNN	RMSSD	HF
**Age**	*TMD*	Pearson’s r *p*-value	−0.71 ** 0.002	−0.57 * 0.021	−0.54 * 0.033
	*Control*	Pearson’s r *p*-value	−0.36 0.19	−0.30 0.27	−0.38 0.16

TMD: temporomandibular disorder; SDNN: standard deviation of all normal RR (NN) intervals; RMSSD: root mean square of successive differences between normal heartbeats; HF: high frequency. HRV indices were transformed in natural logarithms. * *p* < 0.05; ** *p* < 0.01.

**Table 3:** Linear regressions of cognitive performance.

	Predictor	F	η^2^_p_	β	*p*
***Stroop Effect***	**RMSSD**	16.04	0.61	1.11	<0.01
	**Pain Intensity**	11.15	0.53	0.43	<0.01
	**RMSSD × Pain Intensity**	1.60	0.14	−0.30	0.24
					
	**R^2^**	0.84			
	**F**	17.6			
	***p***	>0.001			
***False Alarms***	**RMSSD**	0.52	0.05	0.47	0.48
	**Pain Intensity**	0.81	0.08	−0.27	0.39
	**RMSSD × Pain Intensity**	0.43	0.04	−0.36	0.53
					
	**R^2^**	14			
	**F**	0.56			
	***p***	0.65			

RMSSD: root mean square of successive differences between normal heartbeats.

## Data Availability

The raw data supporting the conclusions of this article will be made available by the authors on request.
